# Developing radiographer roles in the context of advanced and consultant practice

**DOI:** 10.1002/jmrs.2

**Published:** 2013-02-05

**Authors:** Lisa J Field, Beverly A Snaith

**Affiliations:** Radiology Department, Mid Yorkshire Hospitals NHS Trust, Pinderfields HospitalAberford Road, Wakefield, WF1 4DG, U.K

**Keywords:** Advanced practice, bone densitometry, dual-energy absorptiometry, professional boundaries, radiographer reporting, skill mix, ultrasound

## Abstract

Skill-mix initiatives have provided opportunities for radiographers to develop roles and achieve their potential, thus contributing to radiographer retention rates and increased job satisfaction. This reflective article explores two radiographic roles within an interprofessional context including the implications for confidence, competence, and future sustainability. These were reporting roles which extended into two modalities, one into bone densitometry and another into ultrasound. This article discusses how successful skill mix can benefit the individual, their department, and NHS organization and that role expansion can develop a more dynamic and resourceful workforce with transferability of skills and attributes.

## Introduction

In 2007, the Board of the Australian Institute of Radiography (AIR) established the Advanced Practice Working Group with the overall aim of defining an Australian “advanced practitioner model.”^1^ More recently, the work evolved into a larger interprofessional review with an independent chair and a final report that identified a number of recommendations for the professions concerned.^2^ The drivers were seen as technical advances, changing profiles of health-care workers, an ageing population with a growing incidence of cancer, and a health-care sector under increasing financial pressure.

It is acknowledged that the United Kingdom (U.K.) skill-mix model is viewed as a template internationally^2^; however, countries need to identify clinical service and education needs based on their own population and health economy. Skill mix in the U.K. context includes both advanced and consultant roles, together with the delegation of limited scope examinations to an assistant level. Evidence-based examples of advancing practice can provide a useful discussion point for exploring local opportunities and demands, thereby providing an analysis of training needs.

Most U.K. radiography role development has occurred through the adoption of radiological tasks^3^ and the catalyst for this was exponential growth in demand for imaging services, compounded by a shortage of radiologists^4^ and the challenge to reduce waiting times, provide timely reports, and streamline patient pathways.^5^ Changes in radiographer roles may include the extension of scope of practice, with resultant increased accountability and responsibility, or may be an expansion of skills and professional attributes at the same level.^6^

According to the U.K. professional body, the College of Radiographers, skill mix within imaging implies the utilization of specialist skilled radiographers (and others) to complement or increase the expertise available to patients and provides both better patient care and financial savings.^7^ Such initiatives in turn provide opportunities for radiographers to develop new skills and achieve their potential, thus improving job satisfaction and retention rates.

A number of publications have demonstrated how radiographer roles are improving patient pathways and delivering increased capacity; however, the fundamental process of developing and sustaining these initiatives is often poorly evidenced. This reflective article aims to share the experience of a U.K. multi-site National Health Service (NHS) organization in expanding the role of the radiographer to provide an understanding of the opportunities and challenges such developments can bring to the individual, the employer, and the profession. Radiographer role development is well established in the U.K. and had been introduced in the described organization over 15 years ago. Today, radiographers and sonographers work alongside radiology colleagues in a multi-disciplinary team, collectively acquiring and reporting in excess of 380,000 examinations per year. Education and audit programmes together with appropriate guidelines and protocols provide a governance framework for role development, enabling new service initiatives to be explored in a practical and evidence-based way.

This commentary article reflects upon individual radiographic roles in an interprofessional context within general radiography, bone densitometry (dual-energy x-ray absorptiometry [DXA]), and ultrasound services, and discusses the drivers, opportunities, and challenges faced. The two individuals described (the authors) are experienced radiographers with an established advanced practice background in general radiography, including the independent reporting of musculoskeletal and visceral radiographs. Although these are (or aspiring to be) consultant radiographers, the principles and experiences are transferable to other levels of radiographic practice.

## Role 1: Bone Health and Osteoporosis

Within the organization, DXA was a relatively immature service, only initiated 4 years previously, with images acquired by radiographers and reported by physicians external to radiology, including rheumatologists, orthogeriatricians, and endocrinologists. In many organizations, DXA is located outside the umbrella of imaging services, both physically and organizationally, with varying involvement of radiographers in the day-to-day running of the service and often run autonomously by nurse practitioners.^8^

As a result of increasing referrals and service development, there was a need to expand capacity for both image acquisition and in particular reporting. As radiographer role development was well embedded in other imaging modalities, it seemed natural to explore opportunities in DXA. However, even the relatively small size of the service required more than one individual to enable annual leave and sickness cover, but did not justify either practically or financially the development of two advanced practitioner posts. Therefore, through internal application, an established advanced practitioner from general radiography was selected to work alongside a DXA radiographer to establish a radiographer-led reporting service.

In many centres, DXA results comprise numerical scores and categorization of normal, osteopenia, or osteoporotic bone density. In the U.K. guidance states that advice regarding treatment and lifestyle should also be included in order to provide referrers with a holistic service.^9^,^10^ To ensure comparability with the non-radiologist physician reporters, this level of detail was essential, but identified a larger knowledge and skill gap than initially anticipated. This gap was bridged by formal postgraduate education and clinical experience – recognizing that this role was really to sit at an advanced practice level.

The independent reporting of DXA scans at this level by radiographers is still relatively rare,^9^ but is an area for potential further development given the international ageing population.^10^ The role of the diagnostic radiographer in health promotion has also been limited to date; however, this remains within the scope of radiographic practice^11^ and fulfils the aspirations of the 10 key roles for allied health professions in the U.K.^12^

Role development and the delegation of tasks within U.K. imaging centres are well established with recognized clinical supervision and management structures, but this is not replicated externally where cross-disciplinary roles and responsibilities are required. This presented a new challenge as professional respect and capabilities have been established through years of evidence within imaging, but had to be earned within an accelerated timeframe with other clinical groups. Although perceived as a clinical expert, the commencement of reporting in a clinical area not governed by the imaging service was a new venture. Role expansion into DXA meant gaining the professional confidence and credibility of a new peer group whom have had limited experience of allied health professional role development, having worked predominantly with nurses. Although this could be perceived as an opportunity and pushing back the boundaries of multiprofessional working, at times, the process was both personally and professionally demanding. Many of the stresses were due to the lack of clearly defined requirements, which with hindsight should have been developed at the start; however, when initiating new clinical roles and services unexpected issues evolve and need subsequent change.

The initial apprehension felt by the supervising physicians regarding radiographers issuing DXA reports was resolved following successful completion of a postgraduate programme, an audit of competency, and involvement in multidisciplinary team meetings. Professional recognition and credibility is developed over a period of time, and there are perceivable changes in attitude towards advanced radiographic practitioners reporting DXA scans and recommending treatment options. Due to recently published U.K. guidance on the treatment of osteoporosis in the elderly, referrals from primary care to the DXA service are anticipated to substantially increase. Therefore, the impact such roles can have are not only at an imaging service level but include economic savings locally and within the wider NHS, reducing the risk of future fractures due to early diagnosis and treatment.

## Role 2: Emergency Ultrasound

For an experienced consultant radiographer, ultrasound role development was by accident rather than design and arose through the establishment of an emergency department (ED) ultrasound service. ED ultrasound is now an expectation of international emergency care^13^ and is performed by physicians in the trauma and acute settings. These physicians have gained acceptance from radiologists and sonographers in the performance of focused ultrasound examinations with a single diagnostic question.^14^,^15^ As lead radiographer for emergency care, the initial involvement was as facilitator between ED and imaging around acceptance, equipment, and training. However, through discussion and association, the opportunity to develop the same skill set as the ED staff was embraced.

Despite no prior ultrasound experience, this role expansion appeared a natural transition, with a number of years providing immediate definitive reports to the ED and previous education in clinical assessment. After a 2-day training course alongside physicians and a short period of clinical experience in the ED, independent scanning for abdominal trauma or suspected aortic aneurysm commenced when either ED physicians were busy or as a radiographer member of the trauma team. Because of accessibility and familiarity, the physicians approached regularly for advice and ongoing clinical education, but capability to provide more than peer support was limited because education and experience was at the same level. Despite the autonomy of the consultant radiographer role, the focused nature of emergency ultrasound in this context falls short of the practice expected of an advanced practitioner sonographer. Rather, it is the level and scope of other clinical (reporting) and non-clinical activities (education, research, leadership) that justify the “advanced” or “consultant” title.

The transition from expert radiographer to competent sonographer (while maintaining the radiographic role) provided many challenges, particularly in relation to the development and maintenance of ultrasound skills in practice. Student sonographer colleagues perceived the transition to sonographer to be most demanding in relation to the medico-legal aspects and reporting. However, these factors were irrelevant as reporting has the same legal implication regardless of modality and was already autonomous, both within general radiography and limited ultrasound.

As an individual perceived by others as an expert in their field, the practical implications of the disparity in expertise between roles and tasks may cause confusion. Such issues are exacerbated where expectations are raised because of prior (or parallel) interactions at this level, both from staff and patients. This was resolved after a period of time when the desire to consolidate the skills led to completion of a postgraduate diploma in ultrasound with its pre-requisite practical experience and assessments. Appropriately, this placed ultrasound at the advanced practitioner level alongside full-time sonographers, although the speed at which this develops to consultant level, if ever, will depend on exposure and commitment.

Rather than mimicking the role of a consultant sonographer, the resultant role is unique, blending the clinical skills of an emergency care radiographer, reporter, and sonographer. Historically, the only individuals with this multimodality scope were radiologists, and this may be seen to challenge their position and profession. However, precedents have been set and this replicates the role of U.K. consultant mammographers^16^ and their experience across mammography and ultrasound, although in breast imaging the anatomical scope is limited but does involve intervention. Although intervention may appear not to be relevant in emergency care, it does have potential, particularly in the assessment and removal of foreign bodies and this may be an area that merits exploration.

## Discussion

Although a number of examples of radiographer advanced practice have been published,^3^,^6^ few explore the expansion of skills and competencies of individuals already in established advanced roles. Both individuals have encountered difficulties and challenges from a personal and professional perspective; however, there is perceived overall service benefit with increased capacity and improvement in patient pathways and multiprofessional communication. The holistic approach to the role has allowed the radiographer autonomy in clinical decision making, including referral for other imaging or investigations. Although both roles have many similarities, they are different in their origin and interdisciplinary involvement. Role 1 was initiated by the imaging department as a strategic decision to expand the DXA service, and therefore, the development was already supported internally. In contrast, role 2 was developed by the individual who identified an opportunity through professional alliances with another clinical service. This meant that the expansion of skills into ultrasound was openly encouraged by the ED physicians with their long history of engagement with radiographers, whereas radiographer reporting in DXA was a new venture and necessitated working with a group of physicians who had limited experience with radiography role development.

Medical resistance has been previously cited as a potential barrier to radiographer role development, whether through entrenched hierarchies^17^ or perceived lack of underpinning clinical knowledge.^18^ These concepts provided additional challenges to the development of radiographer DXA reporting, but interestingly were not an issue within the ED, possibly because of the longstanding close working relationship of ED physicians and radiographers. It should be acknowledged that despite national agreement, these issues might hinder Australian radiographer role development as a result of inherent professional boundaries.

In the U.K., radiographers have traditionally specialized in an individual modality or patient group, particularly at an advanced level. Increasingly patient pathways have provided the opportunity for definition of new multimodality roles in areas such as breast or gastrointestinal imaging. However, with this, there may be limitations in knowledge and scope, and like the roles described above, responsibility for image acquisition and other operational aspects of these services may sit outside the scope of these roles. The debate around skill mix in departments is often around the philosophy of having radiographers with very “specialist skills” in one area versus the radiographers who can practice a number of areas to meet service demands.

In reality, role development is often initiated, or limited, by external political influences (e.g. waiting lists, staff shortages, and budgets) and internal departmental factors (e.g. management philosophical approach to skill mix and existing staff skills). To ensure flexibility and value for money advanced practice is a necessity in developing a dynamic workforce fit for the future. This raises questions as to whether role development should be allowed to grow infinitely or whether there should be clear boundaries and restrictions. The U.K. radiographic scope of practice suggests that relevance of the new roles is key and should not dilute the core skill set of a radiographer.^11^ In contrast, the traditional picture of the radiographer participating in only a small part of a patient pathway (i.e. taking the x-ray) is long gone and represents a historical stereotypical image. The radiographer's role is changing and may include performing other imaging procedures, advising clinicians on abnormal findings and/or intervention in patient management or treatment. These represent a significant additional responsibility and technical competencies beyond those expected at the point of professional registration and are rightly termed advanced practice.^6^

Themes relating to isolation and lack of support remain an issue when individuals undertake “pioneering roles,”^19^ particularly outside of traditional imaging arenas. Resistance and role conflict may challenge the individual, their manager, and clinical colleagues when developing credible advanced practice roles, especially where there are “turf and territory” issues.^20^,^21^ This includes role developments based solely within imaging, where U.K. experience suggests that a lack of clearly demarcated professional boundaries remain a potential issue for radiologists.^22^ While there is a need to embrace the diversity of new roles, there is also a requirement for awareness of the true identity of each profession and respect for individual views and beliefs.

Staffing in U.K. imaging services accounts for approximately 60% of the total budget expenditure, so this resource must be used effectively, and changes must demonstrate measurable improvements to patient care and services.^7^ Creating different ways of using the talents and skills of the workforce can allow staff to work across traditional professional boundaries,^11^,^12^ and many organizations have maximized the impact of role developments by appropriate staff utilization and expanded or extended their scope to meet service demands. However, there is evidence from the U.K. that radiographers in advanced roles are underutilized,^23^ although it is unclear whether this is because of resistance from medical staff, limited need, or an inability to release capacity due to a shortage of radiographers. The initial rapid uptake of extended roles in the U.K. appears to be slowing, but the proposed changes in Australia with the development of new roles need to be monitored to evaluate future workforce profiles, educational needs, and service trends.

## Conclusion

These U.K.-based examples have demonstrated that successful skill mix can benefit the individual and employer and in turn can develop a more flexible, motivated, and resourceful workforce. These roles are dynamic, evolving to fit the needs of the imaging service but require short- and long-term service, education, and workforce planning to enable sustainability.

Although many individuals will view such innovative radiographer roles positively, others (including radiographers) may display anxieties with regard to a loss of professional identity and the blurring of professional boundaries. Despite the uncertainties associated with this change, opportunities can emerge for radiographers wishing to embrace a professional challenge and develop a new career focus. Australia now needs to consider how such roles may be integrated within its own unique health and political culture and be prepared to grasp the opportunities and aspirations of an expectant profession and the needs of the expanding health-care system.
